# Characterization of genome-wide variations induced by gamma-ray radiation in barley using RNA-Seq

**DOI:** 10.1186/s12864-019-6182-3

**Published:** 2019-10-29

**Authors:** Cong Tan, Xiao-Qi Zhang, Yin Wang, Dianxin Wu, Matthew I. Bellgard, Yanhao Xu, Xiaoli Shu, Gaofeng Zhou, Chengdao Li

**Affiliations:** 1 Western Barley Genetics Alliance, College of Science, Health, Engineering and Education, Murdoch University, Murdoch, WA 6150 Australia; 2 Western Australian State Agricultural Biotechnology Centre, College of Science, Health, Engineering and Education, Murdoch University, Murdoch, WA 6150 Australia; 3 IAEA Collaborating Center, State Key Laboratory of Rice Biology, Zhejiang University, Hangzhou, 310029 China; 4 eResearch Office, Queensland University of Technology, Brisbane, 4000 Australia; 5 Hubei Collaborative Innovation Center for Grain Industry, Yangtze University, Jingzhou, 434023 Hubei China; 6 Western Australia Department of Primary Industry and Regional Development, South Perth, WA 6151 Australia

**Keywords:** Artificial mutagenesis, Dwarf mutant, Gamma-ray radiation, Mutation pattern, RNA-Seq, Single nucleic polymorphism

## Abstract

**Background:**

Artificial mutagenesis not only provides a new approach to increase the diversity of desirable traits for breeding new varieties but are also beneficial for characterizing the genetic basis of functional genes. In recent decades, many mutation genes have been identified which are responsible for phenotype changes in mutants in various species including *Arabidopsis* and rice. However, the mutation feature in induced mutants and the underlying mechanisms of various types of artificial mutagenesis remain unclear.

**Results:**

In this study, we adopted a transcriptome sequencing strategy to characterize mutations in coding regions in a barley dwarf mutant induced by gamma-ray radiation. We detected 1193 genetic mutations in gene transcription regions introduced by gamma-ray radiation. Interestingly, up to 97% of the gamma irradiation mutations were concentrated in certain regions in chromosome 5H and chromosome 7H. Of the 26,745 expressed genes, 140 were affected by gamma-ray radiation; their biological functions included cellular and metabolic processes.

**Conclusion:**

Our results indicate that mutations induced by gamma-ray radiation are not evenly distributed across the whole genome but located in several concentrated regions. Our study provides an overview of the feature of genetic mutations and the genes affected by gamma-ray radiation, which should contribute to a deeper understanding of the mechanisms of radiation mutation and their application in gene function analysis.

## Background

In the past century, thousands of new crop varieties have been bred from induced mutants and cultivated worldwide [1]. These varieties have played an important role in offering desirable agronomic traits including higher yield, improved quality and abiotic stress tolerance. New varieties derived from gamma-ray radiation mutagenesis have accounted for up to 64% of all radiation-induced varieties released from 1930 to 2004 [2]. For instance, ‘Calrose 76’, generated by gamma-ray radiation, was the dominant rice variety grown in California (the United of States) until the late 1970s. ‘Calrose 76’ is about 0.25 m shorter and has higher potential yield than its parent line ‘Calrose’ [3, 4]. Meanwhile, artificial mutants have been widely used to characterize the genetic basis of functional genes controlling complex agronomic traits with great importance in crops [5–9]. For example, research showed that the phenotype change from ‘Calrose’ to ‘Calrose 76’ was due to a single nucleotide substitution induced by gamma-ray radiation in the exon 2 of the rice ‘green revolution gene’ sd-1, which encodes a gibberellin 20-oxidase [4]. While mutants are widely used in breeding and genetic research, the molecular feature and underlying mechanisms of artificial mutations remain unclear.

The most widely used mutant-developing techniques include insertion mutagenesis, chemical mutagenesis and physical mutagenesis [10]. The various mutagenesis technologies have diverse mechanisms and mutation patterns. A comprehensive analysis of flanking sequence tags isolated from a transfer DNA (T-DNA) insertion library, including 31,443 independent transformants, identified that T-DNA insertions were a non-random distribution across the whole genome and occurred more frequently in the distal ends and less so in the centromeric regions [11–13]. Compared with insertion mutagenesis, chemical mutagenesis, e.g., ethyl methane-sulfonate (EMS), can induce high-density mutations with random distribution [10]. It has been suggested that EMS causes mispairing through the chemical modification of nucleotides and mostly results in the transition of C/G to T/A via C-to-T changing [14–16]. It has also been shown that EMS can result in nucleotide deletion or insertion as well as chromosome fracture [17]. Ionizing radiation is prone to causing chromosome alterations such as deletions and inversions, which is a consequence of repairing ionizing-radiation-induced damage [18, 19]. Recently, a study on six rice mutants induced by gamma-ray radiation using whole-genome resequencing found that the mutations were more or less evenly distributed across each chromosome [20, 21]. So far, the feature of mutations induced by gamma-ray radiation in barley has not been characterized. With the rapid advancement of technologies in DNA sequencing and molecular biology, it is possible to investigate different mutation types at the whole-genome level in single nucleotide resolution [22–24]. Bulk segregation analysis and restriction site associated DNA sequencing are effective for identifying mutant genes [25–27]. Direct genome sequencing and comparative analysis have also been used to identify causal genes in individual mutants [24] or to characterize all mutations in a large mutant library [22, 23]. Despite the decreasing cost and advancement of genome sequencing technology, characterizing large and complex genome, such as barley (about 4.83 Gb) [28, 29], still poses a serious challenge. In contrast, whole-transcriptome analysis with total RNA sequencing (RNA-Seq) reveal all coding genes and also multiple forms of noncoding RNAs, offering a cost-effective alternative to the whole-genome sequencing for investigating genetic variants in coding regions, in which mutations are likely to lead to the change of phenotype.

In this study, we used whole-transcriptome sequencing to identify genetic mutations in transcription regions which were induced by gamma-ray radiation in a mutant derived from the barley cultivar Vlamingh. We anchored these gamma-ray radiation mutations into annotated gene models, evaluated their genetic effects on the gene-coding products, and further investigated the pattern of mutation distribution along chromosomes and the base changes in these genetic mutations. Finally, we predicted the potential functions of genes with coding product changes based on protein sequence similarity. Overall, this study aims to provide insights into the mechanism of mutations induced by gamma-ray radiation, so as to accelerate the effective application of radiation mutagens in exploiting important genetic resources and improving crop breeding.

## Results

### SNPs and InDels screening through transcriptome sequencing

**Fig. 1:**
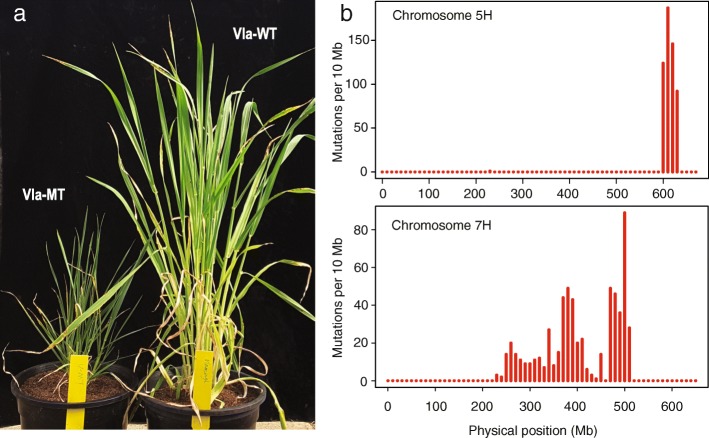
The phenotype of Vla-WT and Vla-MT and distribution of mutations in chromosome 5H and chromosome 7H. **a**, overview of the phenotype of wild-type Vla-WT (right) and mutant type Vla-MT (left). **b**, distribution of detected mutations in chromosome 5 and 7. The x-axis indicates the physical coordination in chromosome 5 and Y-axis indicates the mutation numbers for each 10 Mb region

To understand the feature of genetic mutations induced by gamma-ray radiation, RNA-Seq was performed to investigate the mutations in a gamma-ray radiation mutant (Vla-MT) with dwarf phenotype (Fig. 1a). Vla-MT was derived from the barley cultivar Vlamingh (Vla-WT) irradiated with 200 Gy gamma rays of around 46,000 barley grains and selected from 10,000 individuals of M2 generation. We focused on genetic mutations in coding regions because most mutant phenotypes were reported to be caused by mutations occurred in the coding region. Two biological replicates of both Vla-MT and Vla-WT were sequenced to avoid negative variants and assess the accuracy of transcriptome sequencing and variant detection.

Raw sequencing data of each sample were first filtered and quality-checked before further bioinformatic processing. As a result, an average of 4.67 Gb high-quality clean data was retained for each sample, which represents 78.41 folds of total length (59.56 Mb) of 39,733 representative transcripts of all predicted high-confidence genes in barley [28]. The detail information about the data output for each sample was given in Table 1. The clean reads of each sample were aligned to the barley reference genome with the annotated high-confidence genes as guidance. An average of 95% of the clean reads in each sample was mapped to the barley Morex reference genome [28], of which ~ 89% had unique alignment positions in barley chromosomes. Only the uniquely aligned reads were used to detect SNPs and short InDels for each sample. In theory, in two replicates of each sample, the variants relative to the barley reference should be the same if the sequencing errors and negative-called variants could be avoided. The concordance rate for SNP and InDel calling was 99.98% (45,637 of 45,645) for two replicates of Vla-MT and 99.99% (46,582 of 46,585) for two replicates of Vla-WT, indicating that the potential sequencing errors and false positive variant calling were adequately controlled (within 0.01–0.02%), and the quality of detected variants was reliable for further investigation of genetic mutations induced by gamma-ray radiation in the mutant.

**Table 1:** Reads count in the process of filtration and alignment

Sample (replicate)	Raw Reads		Clean Reads		Uniquely Mapped Reads	
	(n)	(bp)	(n)	(%)	(n)	(%)
Vla-WT (1)	54,869,632	90	51,817,980	94	46,118,002	89
Vla-WT (2)	55,107,192	90	52,013,200	94	46,291,748	89
Vla-MT (1)	54,344,828	90	51,385,788	94	45,733,351	89
Vla-MT (2)	54,698,866	90	51,755,362	94	46,062,272	89
Total	219,020,518	90	206,972,330	–	184,205,373	–

### Genetic mutations induced by gamma-ray radiation

Vla-MT was derived from Vla-WT by exposing it to gamma-ray radiation. Hence, the sequence differences between Vla-MT and Vla-WT are assumed to be genetic mutations induced by gamma-ray radiation. We aligned the transcriptomic sequences from both Vla-MT and Vla-WT to the Morex reference genome sequence; 97% (42,686 of 43,879) of the variants were identical between Vla-MT and Vla-WT. In the two repeats of Vla-MT and Vla-WT, 1193 variants were consistently detected and assumed to be genetic mutations induced by gamma-ray radiation (Additional file 1).

The 1193 genetic mutations induced by gamma-ray radiation were not evenly distributed in each chromosome (Table 2). Instead, these mutations were mainly located on chromosomes 5H (550 mutations) and 7H (612 mutations), with only three mutations on chromosome 1H and one each on 2H, 4H, and 6H. There was no mutation identified on chromosome 3H. Further analysis showed that the genetic mutations on chromosome 5H and 7H were concentrated in several regions (Fig. 1b). On chromosome 5H, the 550 genetic mutations occurred in a 40 Mb genome region (chromosome 5H: 590–630 Mb). On chromosome 7H, the mutations spread across a region of 290 Mb (chromosome 7H: 230–520 Mb). The results suggest that mutations caused by gamma-ray radiation in Val-MT occurred in certain regions of some chromosomes, other than distributed evenly across the whole genome.

**Table 2:** Genetic mutation counts in each chromosome

Chromosome	Length (Mb)	Variants
1H	558.54	3
2H	768.08	1
3H	699.71	0
4H	647.06	1
5H	670.03	550
6H	583.38	1
7H	657.22	612
Un	249.77	25
Total	4833.79	1193

Of the 1193 mutations induced by gamma-ray radiation, 96.65% were SNPs, with the remaining 26 as short deletions and 14 as short insertions. The exact number of base changes is shown in Table 3. Four dominant changes were A➔G, C➔T, G➔A, T➔C.

**Table 3:** Base changes of SNPs revealed as mutations

Base changes	>A	>C	>G	>T	Total
A>	0	46	192	25	263
C>	57	0	66	208	274
G>	211	64	0	33	308
T>	38	170	43	0	251

### Genes affected by mutations

**Fig. 2:**
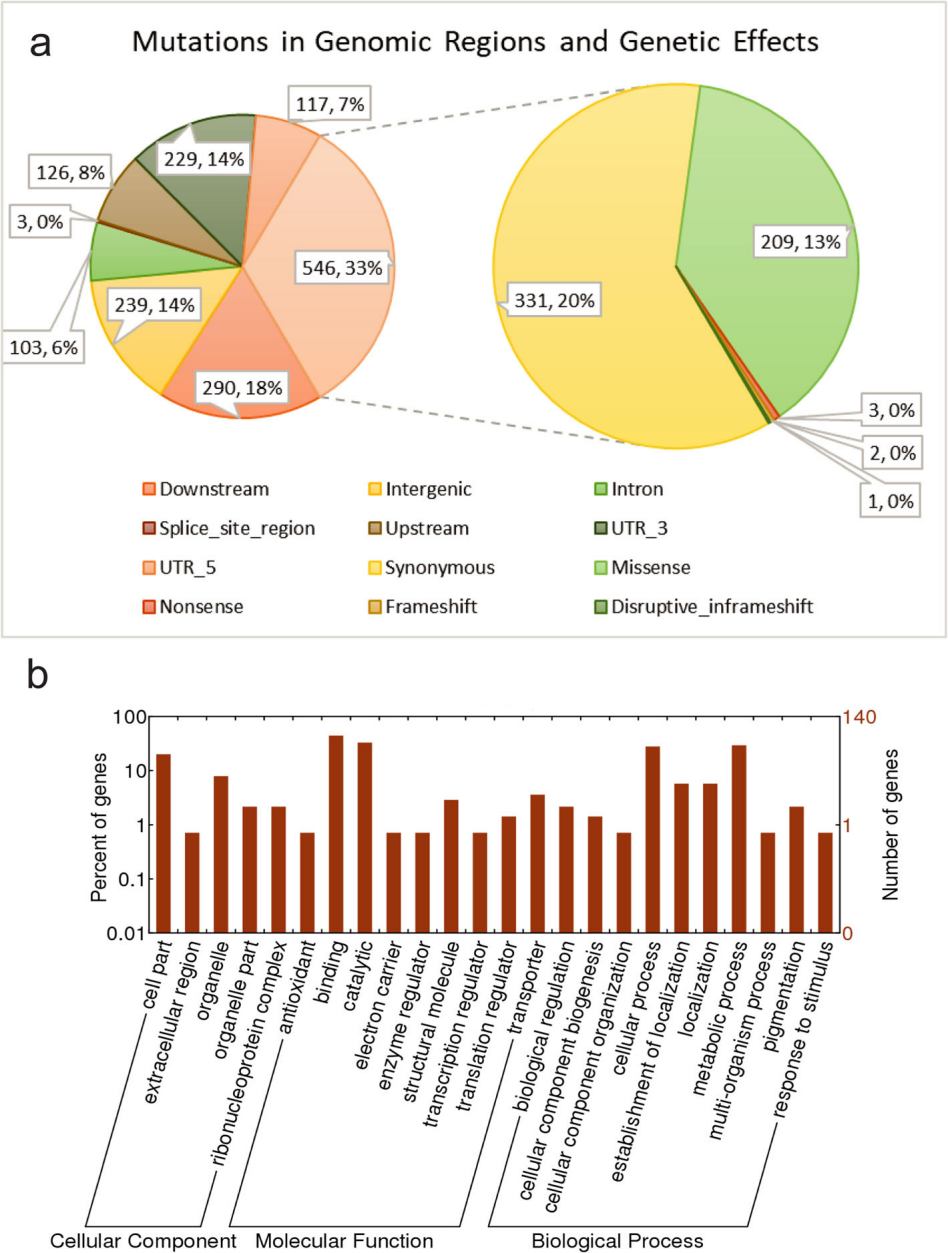
Genetic effects of mutations and biological function of genes with mutant genes. **a**, genetic effect prediction of all detected mutations: left pie chart shows the percent of mutations anchored into different types of genomic regions and right pie chart shows the percent of genetic effects of mutations anchored in exon regions. **b**, biological function classification of affected genes in the cellular component, molecular function, and biological function levels. The x-axis indicates the different types of functions and y-axis indicates the number and percent of genes in each biological function classification

We investigated where the genetic mutations were located by comparing their physical positions with predicted gene models. In theory, they should all be anchored to transcription regions because only mRNA fragments were selected to conduct sequencing in this study. It is interesting to notice that 14% of the mutations were mapped to the intergenic regions and a further 6% to the intron regions (Fig. 2a). It is likely that some potential gene-coding regions were not included in the Morex gene annotations or that alternative splicing events occurred between different germplasm accessions. A further 33% of the detected genetic mutations were anchored to gene exon regions, with the remaining being in upstream, 5′-UTR, 3′-UTR and downstream regions (Fig. 2a, Additional file 2). We further predicted the genetic effects caused by genetic mutations anchored in gene regions based on coding product changing. Figure 2a (pie chart on the right) shows 209 missense mutations, three nonsense mutations, two frameshift mutations, and one disruptive frameshift mutations. The remains are synonymous mutation, which are assumed to cause fewer effects to coding products.

The 215 genetic mutations including missense, nonsense, frameshift, and disruptive frameshift are assumed to be high-level mutations, which are predicted to be within the exon regions to change coding products. These 215 mutations are harbored in 140 out of 26,748 expressed genes (Additional file 3). According to the protein sequence similarity with other well-researched proteins, these 140 genes are annotated with gene ontology. Their functions are classified into three levels being biological processes, molecular function, and cellular components (Fig. 2b). In the biological process level, cellular and metabolic processes dominate the high-level mutation genes, followed by cellular localization and biological regulation. At the molecular function level, binding and catalytic are the two main types of mutation genes. 40 out of the 140 genes with high-level of mutations searched protein hits in KEGG database. According to the KEGG pathway analysis, the 40 hits present in seven major categories of pathways as Additional file 4. The predominant pathway category for the genes with high-level mutations is metabolic pathways, which is followed by biosynthesis of secondary metabolites and biosynthesis of antibiotics.

## Discussion

Most reported mutants have exhibited changes in qualitative traits such as the single tiller mutant [30] and never flowering mutant [31] in rice and six-rowed mutant in barley [32]. The focus in those studies has been on understanding the gene controlling the target trait. Vla-MT, with increased tillers and reduced plant height (Fig. 1a), was initially the selected qualitative mutant. In this study, we found up to 1193 genetic mutations detected in gene transcription regions of the barley mutant generated by gamma-ray radiation. Moreover, they were concentrated on two major chromosomes rather than distributed evenly across the whole genome, which is in contrast with the previous report [20]. This may be due to the fact that our study just focused on a specific mutant. At levels, the mutations may be more or less evenly distributed in the whole genome. The other possibility is that different crop may have different responses to gamma-ray radiation as the size of one barley chromosome is close to the whole rice genome. Further study is required to clarify if the difference is due to a crop-specific response to radiation or is due to the limited number of mutant in our study. Our results also revealed that the genetic mutations in the two chromosomes were within several small fragments of genomic regions. The results raised the question whether it is possible for the two regions as heterozygous in the wild-type Vlamingh but that was excluded after investigating the variants result in the wild-type Vlamingh using whole-genome shotgun sequencing data with high coverage. Our results suggest that a mutant selected from gamma-ray radiation may exist wider genetic variations for other genes and traits. In other words, it may be possible to improve quantitative traits using gamma radiation, as up to 140 genes were modified from a single mutant in the present study.

Apart from the 26 short deletions and 14 short insertions, SNP mutations accounted for about 96.65% of the identified mutations, with A➔G, C➔T, G➔A, and T➔C being the four major types. The chemical groups in the side chain are prone to damage by gamma-ray radiation, which would then be repaired by the DNA repair mechanism [33–35]. It is likely that some mutations could be repaired and recovered to their original chemical structure, while others changed in their similar chemical structures [34]. The scenario could be the same for cytosine (C) and thymine (T) because they share the same monocycle and differ in two chemical groups of the side chain.

Of the 1193 genetic mutations, 450 were anchored in gene exon regions, with 215 predicted to be high-level genetic mutations that cause disruptive in-frameshift, frameshift, nonsense, and missense mutations. The coding products of 140 genes were predicted to be changed by the mutation from gamma-ray radiation. This result contrasts with traditional views that only a few genes are affected and responsible for the mutant phenotype [4–6].

While whole-genome resequencing has been used to identify mutations and isolate mutant genes in species with the simple and small genomes in recent years [22, 23], the sequencing cost for large and complex genomes still pose a challenge. Sequencing depth of 30 folds is recommended for reliable variants analysis [36], therefore 150 Gb data for each sample would be required to investigate mutations in the whole genome for barley [28, 29]. Most variants related to the loss/change function of gene coding product are in the gene-coding regions. Our study with an average data of 4.6 Gb, equivalent to 78.41 folds of the total length of barley gene transcripts, demonstrated that whole transcriptome analysis provides sufficient reliability and resolution as a cost-effective alternative to whole genome sequencing. Meanwhile, whole transcriptome analysis allows the examination of differential expressed genes between mutant and wild plant, which is critical for understanding the genetic and biological basis of mutant genes. Finally, novel transcripts can be detected in transcriptome analysis to improve gene annotation of the reference genome. Variants located in the intron and non-coding regions may be missed in transcriptome sequencing. Although mutations in non-coding regions such as intron region, and intergenic regions, may also play an important role in regulating gene expression level and further leading to phenotype change, the possibility is very low.

Over the past several decades, map-based cloning has played a critical role in identifying genes related to important agronomic traits in crop species and in understanding the genetic basis of plant development in general [37–40]. As molecular marker density has increased dramatically with the application of genotyping-by-sequencing, the current challenge for map-based cloning is population size, which needs to be large enough to produce sufficient recombination events [17, 41]. However, developing a large population to produce sufficient recombination events is laborious and expensive, and is especially challenging for species with large genome size. The present study, through a combination of induced mutation and transcriptome sequencing, provides a complementary strategy to classical bi-parental cross-mapping for the identification of functional genes.

## Conclusion

1. Our study identified that a large number of genes (140) are affected by gamma-ray radiation in a barley mutant.
2. The mutations induced by gamma-ray radiation are not evenly distributed in the whole genome, instead, mutations are located in several concentrated regions in certain chromosomes.
3. Our study provides an overview of the feature of genetic mutations and genes caused by gamma-ray radiation, which should offer a deeper understanding of the mechanisms of radiation mutation and their application in gene function analysis.

## Methods

### Plant materials

Two barley accessions involved in this study are *Hordeum vulgare* L. cv. Vlamingh (Vla-WT) and one Vlamingh mutant (Vla-MT). Vla-WT is a malt barely variety with high yield, bred by Department of Primary Industry and Regional Development Western Australia. Vla-MT was a dwarf mutant induced by 200 Gry ^60^Co gamma radiation from around 46,000 M0 dry seeds of Vla-WT and selected from 10,000 M2 individuals. Vla-WT and Vla-MT were sown in growth pots (diameter 45 cm and 25 cm in height) in the glasshouse of Murdoch University (31.95° S 115.86° E, Perth, Australia) in May 2015 (10.5/13.5, D/N). These plants were grown under normal condition and irrigated twice every week (Monday and Thursday). Leave of both Vla-MT and Vla-WT (with two biological replicates) were collected at six weeks old, frozen in liquid nitrogen immediately and stored at − 80 °C.

### Transcriptome sequencing

Total RNA was extracted using TRIzolTM Reagent (Invitrogen, California, the United States) following its user guide. The integrity and purification of RNA samples were qualified using the 2100 Bioanalyzer instrument (Agilent Technologies, California, the United States) and 1.5% agarose gel. RNA-Seq libraries were constructed following the manufacturer’s instruction of TruSeq RNA Library Prep Kit v2 (Illumina, California, United States) and sequenced on the HiSeq 2000 platform in Beijing Genomics Institute-Shenzhen (Shenzhen, China).

### Reads mapping and SNP/InDel calling

Raw reads (90 bp paired-end) produced by the sequencer were filtered to remove reads with low quality using Sickle [42] (version 1.33, parameters as “pe -q 30 -l 50”). The quality of cleaned data was assessed using FASTQC toolkit [43]. Clean reads of each sample were mapped to the latest barley reference genome [28] using the sliced aligner STAR [44] (version 2.5.3, default parameters). PCR duplications were filtered using SAMtools [45] (version 1.4.1, default parameters). Only reads with unique mapping positions in the reference proceeded to call SNPs and InDels for each sample. This step was performed using the pipeline consisting of SAMtools [45] (version 1.4.1, parameters as “samtools mpileup --adjust-MQ 50 --max-depth 100 --redo-BAQ --min-MQ 20 --min-BQ 13”) and BCFtools [46] (Version 1.5, parameters as “bcftools call -v -c -O z”).

SNPs and InDels were filtered using the following criteria: (1) variants within the low-complexity regions (LCRs) were removed; (2) variants with calling quality < 50 and mapping quality < 40 were removed; (3) variants with more than five read numbers supporting the non-reference allele, and a percentage > 20%, were retained; (4) SNPs within 15 bp of the flanking region of an InDel were removed. SNP and InDels filtration was performed using BCFtools [46] (Version 1.5, parameters as “filter -SnpGap 15, IndelGap 15 –e ‘MQ<40’” ) and VCFtools [47] (Version 0.1.14, parameters as “-minQ 50, --minDP 5, -maxDP 100, -minGQ 20”). The detail processing scripts for these analysis are given as Additional file 5.

### Genetic effects assessment and function annotation for mutations

The genetic effects of each mutation were predicted based on their position in gene models and the change of coding product using SnpEff [48] (Version 4.3, default parameters). The impact effect of each mutation was assessed and classified into four levels: high (including frameshift, nonsense), moderate (including missense), modifier (in intron) and low (synonymous). Those genes with high and moderate impact genetic effects were selected for function and pathway annotation. This step was achieved using AutoFACT [49] (Version 3.4, default parameters) based on the homology of their encoding protein with well-researched genes and proteins stored in databases of NR, Swiss-Prot, COG and GO. KEGG annotation mainly includes two steps: (1) extract K numbers by searching the KEGG database with protein sequences through the web server (https://www.kegg.jp/blastkoala/); (2) obtain pathway annotation by mapping the extracted K numbers to KEGG pathway maps using KEGG mapper (https://www.genome.jp/kegg/mapper.html).

## Supplementary information

**Additional file 1:** 1193 variants induced by gamma-ray radiation.

**Additional file 2:** Summary of genetic effects of mutations in affected genes.

**Additional file 3:** Annotation information of 140 affected genes.

**Additional file 4:** KEGG pathway annotation information.

**Additional file 5:** Major commands for RNA-Seq SNP and InDel variants calling.

## Data Availability

Raw data of transcriptome sequencing involved in this study have been deposited in NCBI Sequence Reads Archive with accession number PRJNA392197 and whole genome shotgun sequencing data of cv. Vlamingh with the accession number SRS1936521.

## Abbreviations

COG: Cluster of Orthologous Groups
EMS: ethyl methane-sulfonate
GO: Gene Ontology
InDel: insertion or deletion of bases
KEGG: Kyoto Encyclopaedia of Genes and Genomes
NR: non-redundant protein sequences
RAD-Seq: restriction site associated DNA sequencing
RNA-Seq: RNA sequencing
SNP: single-nucleotide polymorphism
Swiss-Prot: UniProtKB/Swiss-Prot
Vla-MT: dwarf mutant from cultivar barley Vlamingh in this study
Vla-WT: wild type of cultivar barley Vlamingh
